# Prevalence of thermotolerant *Campylobacter* species in dogs and cats in Iran

**DOI:** 10.1002/vms3.117

**Published:** 2018-08-31

**Authors:** Saam Torkan, Behnam Vazirian, Faham Khamesipour, Gabriel O. Dida

**Affiliations:** ^1^ Department of Small Animal Internal Medicine Faculty of Veterinary Medicine Shahrekord Branch Islamic Azad University Shahrekord Iran; ^2^ Young Researchers and Elite Club Shahrekord Branch Islamic Azad University Shahrekord Iran; ^3^ Cellular and Molecular Research Center Sabzevar University of Medical Sciences Sabzevar Iran; ^4^ Student Research Committee Shiraz University of Medical Sciences Shiraz Iran; ^5^ School of Public Health and Community Development Maseno University Maseno Kenya

**Keywords:** *Campylobacter*, cat, dog, PCR, Iran, thermotolerant

## Abstract

*Campylobacter* is considered the most common bacterial cause of human gastroenteritis in the world with *C. jejuni* being regarded as the primary cause of bacterial gastroenteritis. A broad range of other *Campylobacter* species, including *C. coli* have also been implicated in human gastroenteritis. This study sought to isolate, characterize and assess the antibiogram of *Campylobacter jejuni* and *C. coli* from faecal samples obtained from cats and dogs in Isfahan and Shahrekord cities in Iran. Faecal samples were collected from 100 pets comprising of 50 dogs and 50 cats from March 2015 to March 2016; incorporating the four seasons (spring, summer, autumn and winter). *Campylobacter* spp. was isolated by culture, characterized by biochemical tests and confirmed by PCR‐based assays. Antimicrobial susceptibility test was performed by the Kirby–Bauer disk diffusion method, using Mueller Hinton agar. A total of 19 *Campylobacter* isolates among them two *C. jejuni* and one *C. coli* were recovered from dogs and cats’ faecal samples. The prevalence rates of *Campylobacter* spp. were 16.0% (8 out of 50) in dogs and 22.0% (11 out of 50) in cats. The highest (4 out of 16, 25%) *Campylobacter* spp. prevalence among dogs was reported in autumn and the lowest (1 out of 11, 9.1%) in spring, while among the cats, the highest (4 out of 12, 33.3%) *Campylobacter* spp. prevalence was reported in summer and lowest (1 out of 11, 9.09%) in spring. *Campylobacter* spp. isolated from faecal samples obtained from cats and dogs exhibited the most frequent antimicrobial resistance against tetracycline at 81.8% and 87.5%, respectively, compared to all other antimicrobial agents. These results show a low prevalence of *Campylobacter* spp. in faecal samples obtained from pet dogs and cats in Shahrekord and Isfahan cities in Iran. Given the relatively low prevalence of the *C. jejuni* and *C. coli* in pet dogs and cats in Isfahan and Shahrekord cities, it can be assumed that their importance as reservoirs for infection in humans is likely to be limited to the studied cities, but should not be neglected.

## Introduction

Campylobacteriosis is an important, cosmopolitan, gastrointestinal infection of humans caused by a micro‐aerophilic bacterium; *Campylobacter* (John *et al*. 2002; Moyaert *et al*. 2008; Raissy *et al*. 2014; Jonaidi‐Jafari *et al*. 2016). *Campylobacter jejuni* and *C. coli* are considered among the most common causes of bacterial enteritis in humans and various animals worldwide (Rahimi *et al*. 2012; Goni *et al*. 2017). Consumption of contaminated food (mainly poultry), undercooked meat, unpasteurized milk and contaminated water are the most common mode of transmission. Contamination during food preparation has also been reported in some studies (Rahimi *et al*. 2010, 2017; Ommi *et al*. 2017). Infection with *C. jejuni* and *C. coli* may be asymptomatic or associated with some non‐specific clinical signs such as diarrhoea, weight loss and anorexia (Hakkinen *et al*. 2007). *Campylobacter* spp. have been isolated from various domestic and wild animals worldwide with a high incidence reported among poultry and poultry by‐products (Hosseinzadeh *et al*. 2015; Modirrousta *et al*. 2016; Rahimi *et al*. 2017). The bacterium has also been isolated from the environment, including aquatic environments and sewage (Ghane *et al*. 2010). However, the incidence of *Campylobacter* spp. in the environment largely depends on the climatic conditions of the geographical area (Baserisalehi *et al*. 2007).

Repeated contact with pets and livestock can increase the risk of *Campylobacter* infection in humans (Rahimi *et al*. 2017) with dogs and cats serving as potent reservoir of the *Campylobacter* spp. infection to their owners (Rahimi *et al*. 2012). However, studies show that pet dogs and cats have a relatively lower prevalence of *Campylobacter* spp. infection compared to stray ones (Salihu *et al*. 2010; Goni *et al*. 2017). The prevalence of *Campylobacter* spp. infection in dogs and cats is influenced by factors such as age, concurrent infection with other enteric pathogens and antibiotic treatment (Goni *et al*. 2017). Younger animals have a higher risk of *Campylobacter* spp. infection compared to older ones (Holmberg *et al*. 2015).

Microscopic and biochemical assays are commonly employed in the diagnosis of *Campylobacter* spp. in samples. However, these methods have proved inadequate for identification and classification of various *Campylobacter* species. This has led to the development of more sensitive molecular techniques such as the polymerase chain reaction (PCR) whose use has facilitated accurate identification and classification of a variety of *Campylobacter* species in different hosts (Rahimi *et al*. 2017).

A number of studies on *Campylobacter* spp. infection in Iran have focused on poultry (Rahimi & Ameri 2011; Hosseinzadeh *et al*. 2015; Modirrousta *et al*. 2016), cattle (John *et al*. 2002) and pets (Rahimi *et al*. 2012). This study sought to investigate the burden and identity of *Campylobacter* spp. infecting dogs and cats in Isfahan and Shahrekord cities in Iran.

## Material and methods

### Sample collection

A total of 100 fresh faecal samples from cats (*n* = 50), and dogs (*n* = 50) were collected over the four seasons (spring, March to June; summer, June to September; autumn, September to December; and winter, December to March) between March 2015 and March 2016. The samples were stored in separate sterile plastic bags to prevent cross‐contamination and immediately transported to the laboratory in a cooler box containing ice packs.

### Microbiological analysis

The faecal samples were processed immediately upon arrival at the laboratory, using aseptic techniques. Approximately, 5 g of faeces were homogenized in 45 ml of Preston enrichment broth base‐containing *Campylobacter* selective supplement IV (HiMedia Laboratories, Mumbai, India) and 5% (v/v) defibrinated sheep blood. After inoculation at 42°C for 24 h in a micro‐aerophilic condition (85% N_2_, 10% CO_2_ and 5% O_2_), 0.1 mL of the enrichment broth was streaked onto *Campylobacter* selective agar base (HiMedia Laboratories, Mumbai, India) supplemented with an antibiotic supplement for the selective isolation of *Campylobacter* species (HiMedia Laboratories, Mumbai, India) and 5% (v/v) defibrinated sheep blood. The agar plates were incubated at 42°C for 48 h under the same conditions. Presumptive thermotolerant *Campylobacter* colonies from each selective agar plate were subjected to biochemical tests. For identification, standard microbiological and biochemical procedures were used, including Gram staining, production of catalase, oxidase, hippurate hydrolysis, urease activity, indoxyl acetate hydrolysis and susceptibility to cephalotin (Rahimi & Ameri 2011).

### DNA extraction and PCR conditions

From Preston's broth DNA was extracted from samples after the enrichment step, using a Genomic DNA purification kit (Fermentas, GmbH, Germ any, K0512) following the manufacturer's protocol. The PCR method used in this study is similar to the one previously described by Denis *et al*. (1999).

Using this protocol, the three genes selected for the identification of the *Campylobacter* spp., *C. jejuni*, and *C. coli* were the 16S rRNA gene (Linton *et al*. 1997), the *mapA* gene (Stucki *et al*. 1995), and the *ceuE* gene (Gonzalez *et al*. 1997), respectively. The primers sets used were: 16SrRNA (Forward: 5′‐ ATC TAA TGG CTT AAC CAT TAA AC and Reverse: 5′‐ GGA CGG TAA CTA GTT TAG TAT T) for the identification of *Campylobacter* spp., *mapA* (Forward: 5′‐ CTA TTT TAT TTT TGA GTG CTT GTG and Reverse: 5′‐ GCT TTA TTT GCC ATT TGT TTT ATT A) for *C. jejuni* and *ceuE* (Forward: 5′‐ AAT TGA AAA TTG CTC CAA CTA TG and Reverse: 5′‐ TGA TTT TAT TAT TTG TAG CAG CG) for *C. coli*.

Amplification reactions were performed in a 30‐*μ*L mixture containing 0.6 U of Taq polymerase (Fermentas, GmbH, Germany), 100 *μ* mol L^−^¹ of each deoxynucleoside triphosphate (dNTP), 0.11 *μ* mol L^−^¹ of MD16S1 and MD16S2 primers, and 0.42 *μ* mol L^−^¹ of MDmapAl, MDmapA2, COL3 and MDCOL2 primers in the Fermentas buffer (Fermentas, GmbH, Germany). Amplification reactions were carried out, using a DNA thermal cycler (Master Cycle Gradiant, Eppendorf, Hamburg, Germany) with the following program: 1 cycle of 10 min at 95°C, 35 cycles each consisting of 30 s at 95°C, 1 min and 30 s at 59°C, 1 min at 72°C, and a final extension step of 10 min at 72°C. The amplification generated 857 bp, 589 bp, and 462 bp DNA fragments corresponding to the *Campylobacter* genus, *C. jejuni*, and *C. coli*, respectively.

*Campylobacter coli* (ATCC 33559) and *C. jejuni* (ATCC 33560) were used as the positive controls and DNase‐free water was used as the negative control. The PCR products were stained with a 1% solution of ethidium bromide and were visualized under UV light after gel electrophoresis on 1.5% agarose.

### Antimicrobial susceptibility testing

Antimicrobial susceptibility testing was performed by the Kirby–Bauer disk diffusion method using Mueller Hinton agar (HiMedia Laboratories, Mumbai, India) supplemented with 5% defibrinated sheep blood, following the procedure outlined by the Clinical Laboratory Standards Institute (CLSI 2006). The following antimicrobial impregnated disks (HiMedia Laboratories, Mumbai, India) were used: nalidixic acid (30 *μ*g), ciprofloxacin (5 *μ*g), erythromycin (15 *μ*g), tetracycline (15 *μ*g), streptomycin (15 *μ*g), gentamicin (10 *μ*g), amoxicillin (30 *μ*g), ampicillin (10 *μ*g), chloramphenicol (30 *μ*g), enrofloxacin (5 *μ*g), cefazolin (30 *μ*g), and lincomycin (10 *μ*g).

Briefly, well‐isolated colonies of same morphological type were selected from an agar plate culture and transferred into 10 mL of sterile saline buffer (NaCl 0.9%). After homogenization, 2 mL of the mixture were flooded onto the surface of a Mueller‐Hinton agar (Oxoid) containing 5% defibrinated sheep blood. The inoculum was allowed to dry for 5 min and antibiotic discs placed on the plate. After 48 h of micro‐aerobic incubation at 37°C, diameters of the inhibition zones were measured with calipers and the results interpreted in accordance with interpretive criteria provided by CLSI (2006). *Staphylococcus aureus* and *Escherichia coli* were used as quality control organisms in antimicrobial susceptibility determination. The antimicrobial agents tested in this study are widely used to treat infections in people and animals in Iran.

### Statistical analysis

Data generated were subjected to descriptive statistics using Microsoft Excel version 2010 (Microsoft, USA) and expressed in percentages. The association of age, sex and location with the presence of *Campylobacter* were compared by the *χ*² test using the statistical package for social sciences (SPSS) version 26.

## Results

### Prevalence of *Campylobacter* species in dogs and cats

Among faecal samples of 50 dogs that were tested in this study, *Campylobacter* spp. were isolated from 8 samples reflecting a prevalence of 16%, while only one in 8 positive samples tested for *Campylobacter jejuni* accounting for 12.5% of the isolates. However, no *C. coli* was isolated from dogs’ faecal samples in the current study (Table 1). The prevalence (5 of 15, 33.3%) of *Campylobacter* spp. in dogs greater than 1 year of age was significantly higher (*P* = 0.029) compared to dogs aged below 1 year (3 of 35, 8.58%). A higher *Campylobacter* species isolation rate was recorded in male (6 of 30, 20%) than female dogs (2 of 20, 10%), though the difference was not statistically significant (*P* = 0.345). *Campylobacter* spp. isolated from dogs was higher in Shahrekord city (3 of 17, 17.6%) than Isfahan city (5 of 33, 15.2%), while infection was highest during autumn (4 of 16, 25%) and lowest during spring (1 of 11, 9.09%) (Table 1). However, the prevalence of *Campylobacter* spp. infection in dogs did not show any significant differences with regards to location (*P* = 0.820) nor with seasons (*P* = 0.780).

**Table 1:** Prevalence of *Campylobacter* species in dogs in Iran

Parameters	No. sampled	No. positive (%)		
Parameters	No. sampled	*Campylobacter* spp.	*C. jejuni*	*C. coli*
Age				
<1 year	35	3 (8.58)	0 (0.00)	0 (0.00)
>1 year	15	5 (33.33)	1 (6.66)	0 (0.00)
Sex				
Male	30	6 (20.00)	1 (3.33)	0 (0.00)
Female	20	2 (10.00)	0 (0.00)	0 (0.00)
Location				
Isfahan	33	5 (15.15)	0 (0.00)	0 (0.00)
Shahrekord	17	3 (17.65)	1 (5.89)	0 (0.00)
Season				
Summer	13	2 (15.39)	0 (0.00)	0 (0.00)
Autumn	16	4 (25.00)	1 (6.25)	0 (0.00)
Winter	10	1 (10.00)	0 (0.00)	0 (0.00)
Spring	11	1 (9.09)	0 (0.00)	0 (0.00)
Total	50	8 (16.00)	1 (2.00)	0 (0.00)

Among the cats, an overall *Campylobacter* spp. prevalence of 22% (11 out of 50) was reported in the current study. *Campylobacter jejuni* and *C. coli* accounted for 7.69% (1 of 13 positive isolates) each. A higher prevalence of *Campylobacter* spp. was recorded among female cats (8 of 32, 25%) and those cats aged below 1 year (7 of 30, 23.3%), compared to male cats (3 of 18, 16.7%) and cats above 1‐year‐old (4 of 20, 20%). The differences observed in the prevalence of *Campylobacter* between the cats of different age groups (*P* = 0.780) and sexes (*P* = 0.495) were, however, not statistically significant. *Campylobacter* spp. isolated from cats were higher in Shahrekord city (3 of 6, 50%) than Isfahan city (8 of 44, 18.2%). In addition, *Campylobacter* spp. isolates were relatively more during summer and lower during spring, though the differences with regards to locations (*P* = 0.078) and seasons (*P* = 0.082) were not significant (Table 2). Generally, there was no significant difference (*P* = 0.444) in the prevalence of *Campylobacter* infection recorded in dogs and cats in this study.

**Table 2:** Prevalence of *Campylobacter* species in cats in Iran

Parameters	No. sampled	No. positive (%)		
Parameters	No. sampled	*Campylobacter* spp.	*C. jejuni*	*C. coli*
Age				
<1 year	30	7 (23.33)	1 (3.33)	1 (3.33)
>1 year	20	4 (20.00)	0 (0.00)	0 (0.00)
Sex				
Male	18	3 (16.66)	0 (0.00)	0 (0.00)
Female	32	8 (25.00)	1 (3.13)	1 (3.13)
Location				
Isfahan	44	8 (18.18)	1 (2.27)	0 (0.00)
Shahrekord	6	3 (50.00)	0 (0.00)	1 (16.66)
Season				
Summer	12	4 (33.33)	1 (8.33)	0 (0.00)
Autumn	15	4 (26.66)	0 (0.00)	1 (6.66)
Winter	12	2 (16.66)	0 (0.00)	0 (0.00)
Spring	11	1 (9.09)	0 (0.00)	0 (0.00)
Total	50	11 (22.00)	1 (2.00)	1 (2.00)

### Antibiotic sensitivity of *Campylobacter* species in dogs and cats

Multiple antibiotic resistance patterns were observed in the current study. *Campylobacter* species were most frequently resistant to tetracycline (7 of 8, 87.5%), ciprofloxacin (6 of 8, 75%), nalidixic acid (5 of 8, 62.5%), cefazolin (4 of 8, 50%) and amoxicillin (2 of 8, 25%). Overall, *Campylobacter* spp. showed some level of resistance to all antimicrobial agents except gentamicin. However, *C*. *jejuni* isolates from dogs’ faeces showed resistance to five (nalidixic acid, ciprofloxacin, tetracycline, cefazolin and lincomycin) of the 12 antimicrobial agents tested in this study (Table 3). Considering *Campylobacter* strains isolated from cats’ faeces, the largest proportion of *Campylobacter* spp. (9 of 11, 81.8%), *C. jejuni* (10 of 11, 90.9%) and *C. coli* (1 of 1, 100%) were resistant to tetracycline while 72.7% (8 of 11) of *Campylobacter* spp. and 63.6% (7 of 11) of *C. jejuni* were resistant to erythromycin. In addition, *C. jejuni* was highly resistant to nalidixic acid, ciprofloxacin, amoxicillin, enrofloxacin, cefazolin and lincomycin while low resistance was recorded against streptomycin, ampicilin and chloramphenicol. The single *C. coli* isolate from cats’ faeces was sensitive to all the antibiotics tested except nalidixic acid (100%), ciprofloxacin (100%) and tetracycline (100%) (Table 4).

**Table 3:** Antimicrobial resistance profiles of *Campylobacter* strains isolated from faeces of dogs

Antimicrobial agent	*Campylobacter* spp. (%)	*C. jejuni* (%)
Nalidixic acid	5 (62.5%)	1 (100%)
Ciprofloxacin	6 (75%)	1 (100%)
Erythromycin	1 (12.5%)	0 (0%)
Tetracycline	7 (87.5%)	1 (100%)
Streptomycin	1 (12.5%)	0 (0%)
Ampicillin	1 (12.5%)	0 (0%)
Amoxicillin	2 (25%)	0 (0%)
Gentamicin	0 (0%)	0 (0%)
Chloramphenicol	1 (12.5%)	0 (0%)
Enrofloxacin	1 (12.5%)	0 (0%)
Cefazolin	4 (50%)	1 (100%)
Lincomycin	6 (75%)	1 (100%)

**Table 4:** Antimicrobial resistance profiles of *Campylobacter* strains isolated from faeces of cats

Antimicrobial agent	*Campylobacter* spp. (%)	*C. jejuni* (%)	*C. coli* (%)
Nalidixic acid	6 (54.54%)	7 (63.63%)	1 (100%)
Ciprofloxacin	5 (45.45%)	4 (36.36%)	1 (100%)
Erythromycin	8 (72.72%)	7 (63.63%)	0 (0%)
Tetracycline	9 (81.81%)	10 (90.90%)	1 (100%)
Streptomycin	2 (18.19%)	1 (9.09%)	0 (0%)
Ampicillin	0 (0%)	1 (9.09%)	0 (0%)
Amoxicillin	4 (36.37%)	5 (45.45%)	0 (0%)
Gentamicin	0 (0%)	0 (0%)	0 (0%)
Chloramphenicol	0 (0%)	1 (9.09%)	0 (0%)
Enrofloxacin	5 (45.45%)	4 (36.36%)	0 (0%)
Cefazolin	5 (45.45%)	6 (54.54%)	0 (0%)
Lincomycin	5 (63.63%)	4 (36.36%)	0 (0%)

## Discussion

Most investigations concerning campylobacteriosis in Iran have largely focused on poultry and its products (Rahimi & Ameri 2011; Hosseinzadeh *et al*. 2015; Modirrousta *et al*. 2016), with only a few studies focusing on dogs and cats in the country (Rahimi *et al*. 2012). The overall prevalence of *Campylobacter* spp. reported among dogs (16%) and cats (22%) in the current study were similar to those reported by Goni *et al*. (2017) in Malaysia but lower than those previously reported in other parts of Iran and a few other countries (Hald *et al*. 2004; Salihu *et al*. 2010; Carbonero *et al*. 2012). The variations in the prevalence of *Campylobacter* species in dogs and cats have been associated with the locality under study, population of dogs sampled, method of identification and the fastidious nature of the organism (Byrne *et al*. 2007; Goni *et al*. 2017). The detection of *Campylobacter* infection in dogs and cats in the present study and a previous report by Rahimi *et al*. (2012) suggests that the organism may be endemic in parts of Iran, thus implying that the infection could be triggered by various environmental and host factors. This observation, however, requires further investigation to better understand the transmission dynamics of the *Campylobacter* spp. infection in the study area.

The commonly isolated *Campylobacter* species in dogs and cats include *C. upsaliensis*,* C*. *helviticus*,* C. jejuni* and *C. coli* with the predominant isolate being *C. upsaliensis* in dogs and cats. In the present study, only *C. jejuni* and *C. coli* were isolated, with the former being predominant. This observation is similar to findings by Baker *et al*. (1999) and Carbonero *et al*. (2012) who reported higher *C. jejuni* isolates compared to *C. coli* in dogs and cats. Both *C. jejuni* and *C. coli* have also been isolated successfully from environmental sources, faeces and by‐products of various mammals and birds (Rosef *et al*. 1985; Hakkinen *et al*. 2007; Moyaert *et al*. 2008; Goni *et al*. 2017). Nawal (2011) isolated *C. jejuni* from humans and poultry in Egypt implying that a wide range of organisms could be susceptible. The small sample size in the current study may be incriminated for the low incidence of *C. coli* reported among cats and absence of the same in dogs.

Dogs above 1 year of age had significantly higher prevalence of *Campylobacter* infection compared to younger ones in the present study. These findings contradicted those of other researchers who reported higher *Campylobacter* infections in younger dogs than older dogs (Hald *et al*. 2004; Wieland *et al*. 2005; Rahimi *et al*. 2012; Goni *et al*. 2017). Consistent with the current findings, Rahimi *et al*. (2012b) also reported that the age of dogs and cats did not have a significant influence on *Campylobacter* infection.

Seasonal variations of the *Campylobacter* species infection in dogs and cats observed in the current study contradicts the findings presented by Rahimi *et al*. (2012) in which they reported an insignificant association between *Campylobacter* infection and seasons in Iran. Large‐scale epidemiological studies should thus be conducted in dogs and cats in Iran to establish the statistical reliability of seasonality on campylobacteriosis in these animals.

Antibiotic resistance of *Campylobacter* species has been identified as an increasing public health concern (Silva *et al*. 2011; WHO, 2013). This is emphasized by the increasing number of multi‐drug resistant isolates, especially to macrolides and fluroquinolones (Alfredson & Korolic 2007). Tetracycline resistance was most frequent in *Campylobacter* strains isolated from both dogs and cats in this study which corroborates the reports by Rahimi *et al*. (2017), D'lima *et al*. (2007) and Nawal (2011). This is particularly important because cats and dogs represent potential sources of spread of antimicrobial resistance. Increased frequency of antimicrobial resistance often results from the indiscriminate and frequent use of a number of antimicrobials for prophylactic and therapeutic treatment of a wide range of bacterial infections, thus promoting the development of resistance among many bacteria. According to Watson & Rosin (2000), the most frequent causes of antimicrobial treatment in dogs and cats are skin and wound infections, otitis externa, respiratory infections, and urinary tract infections.

In the current study, a high frequency of resistance, ranging from 62.5% to 75.0%, was recorded for ciprofloxacin, lincomycin and nalidixic acid in dogs. These findings are almost similar to previous reports in livestock, poultry and humans in Iran and Poland (Rozynek *et al*. 2008; Kumar *et al*. 2012; Rahimi *et al*. 2017). Furthermore, low levels of resistance were recorded for erythromycin, streptomycin, ampicillin, chloramphenicol, enrofloxacin and amoxicillin in dogs in this study. This was also similar to the resistance patterns reported by Rahimi *et al*. (2017). It, however, contradicts the reports in poultry by Ge *et al*. (2003), Nawal (2011) and Kumar *et al*. (2012). The variations in the antibiogram may be associated with the different animal species studied.

All *Campylobacter* isolates from dogs and cats’ faecal samples were sensitive to gentamicin, which is consistent with the reports of Nawal (2011) and Rahimi *et al*. (2017). The antibiogram of *C. jejuni* in dogs in the current study were similar to those reported by Rahimi *et al*. (2017). The high frequency of resistance observed for some antimicrobial agents in the current study is a source of concern, given the close contact between household pets and humans, which offer favourable conditions for transmission of the bacteria by direct contact (e.g. through petting, licking or physical injuries) or through contact with contaminated household environment like floors and carpets (Tan 1997). Furthermore, children are at a relatively higher risk compared to adults because of their closer physical contact with pets as well as contaminated environments (Salfield & Pugh 1987).

The present investigation showed a low prevalence of *Campylobacter* spp. in both pet dogs and cats. However, the results suggest an age predisposition where older dogs are more likely to shed *Campylobacter* spp. than younger dogs. Though the sample size was considerably low, (*n* = 100), the findings provide an insight into the epidemiology of *Campylobacter* infections in dogs and cats in Isfahan and Shahrekord cities in Iran. Nevertheless, given the relatively low prevalence of the *C. jejuni* and *C. coli* in pet dogs and cats in the current study, it can be assumed that their importance as reservoirs for infection in humans is likely to be limited, but should not be neglected. To establish the zoonotic potential of canine and feline *Campylobacter* isolates, both human and canine/feline isolates have to be further characterized and compared.

## Source of funding

This study did not receive funding from public or private sector agencies.

## Conflicts of interest

The authors certify that there is no conflict of interest with any financial organization.

## Ethics statement

The experimental protocol of the present study was approved by Faculty of Veterinary Medicine and Ethical and Research Committee of Islamic Azad University, Shahrekord Branch, Shahrekord, Iran.

## Contributions

All authors read and approved the final manuscript.
